# Mesothelin CAR‐engineered NK cells derived from human embryonic stem cells suppress the progression of human ovarian cancer in animals

**DOI:** 10.1111/cpr.13727

**Published:** 2024-08-13

**Authors:** Yanhong Liu, Min Zhang, Xiaoyan Shen, Chengxiang Xia, Fangxiao Hu, Dehao Huang, Qitong Weng, Qi Zhang, Lijuan Liu, Yanping Zhu, Lei Wang, Jie Hao, Mengyun Zhang, Tongjie Wang, Jinyong Wang

**Affiliations:** ^1^ Key Laboratory of Organ Regeneration and Reconstruction, State Key Laboratory of Stem Cell and Reproductive Biology Institute of Zoology, Chinese Academy of Sciences Beijing China; ^2^ Beijing Institute for Stem Cell and Regenerative Medicine Beijing China; ^3^ Department of Obstetrics and Gynecology Peking University People's Hospital Beijing China; ^4^ National Stem Cell Resource Center, Chinese Academy of Sciences Beijing China; ^5^ Lead contact

## Abstract

CAR‐NK cell therapy does not require HLA matching and has minimal side effects. However, traditional methods of engineering CARs into human tissue‐derived NK cells exhibit heterogeneity, low transduction efficiency, and high manufacturing costs. Here, we provide a reliable approach for generating large‐scale and cryopreserved mesothelin (MSLN) CAR‐NK cells from human embryonic stem cells (hESCs) as an alternative cell source. We first constructed MSLN CAR‐expressing hESCs to reduce CAR engineering costs and subsequently differentiated these stem cells into MSLN CAR‐NK cells via an efficient organoid induction system. The MSLN CAR‐NK cells exhibit the typical expression patterns of activating receptors, inhibitory receptors, and effector molecules of NK cells. In the presence of tumour cells, the MSLN CAR‐NK cells show increased secretion of IFN‐γ and TNF‐α, as well as elevated CD107a expression level compared with induced NK cells. We cryopreserved the MSLN CAR‐NK cells in liquid nitrogen using a clinical‐grade freezing medium (CS10) for more than 6 months to mimic an off‐the‐shelf CAR‐NK cell product. The thawed MSLN CAR‐NK cells immediately recovered after 48–72‐h culture and effectively eliminated ovarian tumour cells, including human primary ovarian tumour cells from patients. The thawed MSLN CAR‐NK cells efficiently suppressed ovarian tumour development in vivo and prolonged the survival of tumour‐bearing mice. Our study provides insights into the clinical translation of hESC‐derived MSLN CAR‐NK cells as a promising off‐the‐shelf cell product.

## Graphical abstract

We provide a reliable approach for generating large‐scale and cryopreserved mesothelin (MSLN) CAR‐NK cells from human embryonic stem cells (hESCs) as an alternative cell source. The thawed MSLN CAR‐NK cells immediately recovered after 48–72‐h culture and effectively eliminated the ovarian tumour cells in vitro and in vivo.

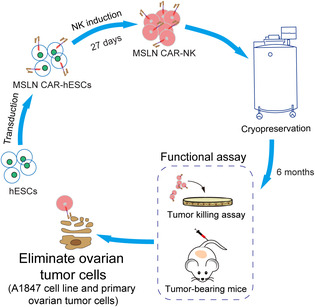

## 1 INTRODUCTION

Natural killer (NK) cells are lymphocytes of the innate immune system, possessing inherited features of killing aberrant cells in a non‐specific manner.^1^ NK cells can lyse the tumour cells directly via perforin or granzyme, and indirectly through cytokines or molecules such as Fas ligand and TRAIL.^2^ Allogenic NK cells exhibit high safety in patients, which makes them a promising alternative to T‐cell therapy.^3^, ^4^ The clinical data have verified that NK cells or CAR‐NK cells have minimal side effects.^5^ However, short‐term persistence in vivo and lack of long‐term treatment efficacy are the key problems in NK cell or CAR‐NK cell therapy.^1^ Therefore, NK cell therapy usually needs high and multiple doses of NK cells, leading to the high costs of manufacturing.

Currently, the NK cells and CAR‐NK cells used for the clinical trials are mainly from peripheral blood cells, umbilical cord blood cells, and NK‐92 cell lines, resulting in variations in NK cell homogeneities. Traditional approaches to generate CAR‐NK cells contain viral transduction (using lenti‐ or retroviruses), or transfection with CAR‐expressing vectors by electroporation.^6^ These methods face problems, such as variations of CAR expression, high manufacturing costs, and limited expansion yields of CAR‐NK cells, which restrict their applications for making off‐the‐shelf CAR‐NK cell products.^7^ Encouragingly, pre‐engineered CAR‐hPSC‐induced CAR‐NK (CAR‐iNK) cells possess off‐the‐shelf potential due to their advantages of unlimited cell source and homogeneity. CAR‐iNK cells or enhanced iNK cells derived from hPSCs exhibit promising antitumor activity against blood and certain solid tumours.^8^, ^9^, ^10^

The development of CAR‐NK cells for treating solid tumours has achieved significant breakthroughs and presents promising advantages.^11^, ^12^, ^13^ Mesothelin (MSLN) is emerging as an attractive therapeutic target for CAR‐NK cell therapy, considering its low expression on normal tissues and high expression in a broad spectrum of solid tumours. Particularly, high expression of MSLN is observed in 60%–65% ovarian cancer.^14^ There have been several clinical trials for ovarian cancer treatment using CAR‐T targeting the MSLN antigen.^15^, ^16^ However, using CAR‐NK cells to target MSLN against ovarian tumours is still in the initial stages.

In this study, we generated the MSLN CAR‐expressing hESCs and induced them into MSLN CAR‐iNK cells. To mimic off‐the‐shelf CAR‐iNK cell products for clinical applications, the harvested MSLN CAR‐iNK cells underwent cryopreservation in liquid nitrogen for more than 6 months. Despite a 20%–30% cell loss 24 h after revival, the total number and viability of MSLN CAR‐iNK cells recovered to over 90% with an extended 24–48 h incubation. The MSLN CAR‐iNK cells efficiently eliminated the MSLN^+^ tumour cells in vitro. Furthermore, the cryopreserved MSLN CAR‐iNK cells significantly suppressed tumour growth and prolonged the survival of tumour‐bearing mice compared to iNK cells. Therefore, our study offers insights into the clinical translation of hESC‐derived MSLN CAR‐iNK cells in the form of cryopreserved off‐the‐shelf cell products.

## 2 MATERIALS AND METHODS

### 2.1 Cell culture

The human ESC (hESC) line was provided by the National Stem Cell Resource Center, Institute of Zoology, Chinese Academy of Sciences. The hESC line was maintained in Essential 8 medium (Gibco) on vitronectin (Gibco) coated plates. The OP9 cell line was purchased from ATCC and cultured with α‐MEM (Gibco) with 20% fetal bovine serum (FBS) (Ausbian). iNK cells or CAR‐iNK cells were cultured in KBM581 medium (Corning) supplemented with IL‐2 (200 IU/mL) and SGR‐SM (1%, DAKEWE) (NK cell medium). Primary human ovarian cancer cells were obtained from Peking University People's Hospital with the donors' informed consent. The primary ovarian cancer cells were cultured in Human Ovarian Carcinoma Cell Medium (Hefei PreceDo Medical Laboratory Co., Ltd). A1847 cell line was purchased from Shanghai Honsun Biological Technology Co., Ltd and cultured in RPMI 1640 Medium (Gibco) supplemented with 10% FBS (Ausbian). AGS cell line was purchased from Wuhan Pricella Biotechnology Co., Ltd and cultured in Ham's F‐12 (Gibco) supplemented with 10% FBS (Ausbian).

### 2.2 Plasmid Construction and electroporation

The single‐chain fragment variable (scFv) specific for mesothelin^17^ was used for CAR construction (MSLN scFv‐CD8 α Hinge‐CD8 α TM‐CD3 ζ). The CAR construct was cloned into the PiggyBac expression vector (PB530A‐2, SBI) which deleted the IRES‐GFP elements to generate PB‐EF1α‐MSLN CAR vector.^8^, ^18^ The MSLN CAR PiggyBac vectors were then transduced into the hESCs by electroporation (Celetrix, 11‐0106). Seven days after transduction, hESCs were stained with PE‐Labelled Human Mesothelin/MSLN (296‐580) Protein, His Tag (Acro, MSN‐HP2H5), and sorted by BD FACSAria™ Fusion for two rounds to establish the stable MSLN CAR‐expressing hESC (MSLN CAR‐hESC).

### 2.3 Generation of MSLN CAR‐iNK cells

The method to induce MSLN CAR‐iNK cells from MSLN CAR‐expressing hESCs was as previously described.^19^ Briefly, MSLN CAR‐hESCs were subjected to a 2‐day monolayer induction to induce highly purified MSLN CAR lateral plate mesoderm cells (MSLN CAR‐iLPM). Subsequently, 2 × 10^4^ MSLN CAR‐iLPM and 5 × 10^5^ OP9 cells were assembled into organoid aggregates and seeded into the transwell (Coring, 3450), establishing an air‐liquid interface for MSLN CAR‐iNK differentiation. Mature MSLN CAR‐iNK cells were obtained after 25‐day induction.

### 2.4 RNA‐seq and data analysis

The MSLN CAR‐iNK cells (CD45^+^CD3^−^CD56^+^MSLN CAR^+^) and iNK cells (CD45^+^CD3^−^CD56^+^) were sorted on day 27 for 10× scRNA‐seq. Droplet‐based scRNA‐seq datasets were produced using a chromium system (10× Genomics, PN120263) following the manufacturer's instructions. Droplet‐based scRNA‐seq datasets were aligned to reference genome GRCh19 and quantified using the CellRanger software package (version 7.0) and subjected to Seurat (version 4.3.0) for further analysis. Projection of MSLN CAR‐iNK cells onto iNK cells was performed using the Seurat package. Before integrating data, we performed subsequent quality control (QC). To pass QC, cells were required to have less than 10% of aligned reads mapping to mitochondrial genes and less than 5% of aligned reads mapping to haemoglobin genes. Then we performed simple linear regression against the cell cycle score calculated by cell cycle scoring to rule out the effects of cell cycle variances. All datasets were integrated using Seurat's integration function. Anchors were identified with the Find Integration Anchors function, and then the Integrate Data function was used with dim = 1:30. The standard workflow for UMAP dimensionality reduction was performed using the top 30 PCs. Violin plots for gene expression were plotted using the VlnPlot function of Seurat and the ggplot2 package. The raw data of MSLN CAR‐iNK cells (fastq files) were uploaded to the Genome Sequence Archive public database (HRA007500). The raw data of iNK RNAseq data were available from the Genome Sequence Archive public database (HRA001609).

### 2.5 Flow cytometry

Cells were blocked by Human TruStain FcX™ (Biolegend, 422,302) antibody, and then stained with related antibodies. The following antibodies or fluorescence labelled proteins were used: CD45 (Biolegend, HI30), CD3 (Biolegend, HIT3a), CD16 (Biolegend, 3G8), CD56 (Biolegend, HCD56), CD244 (Biolegend, C1.7), DNAM‐1(Biolegend, 11A8), NKp30 (Biolegend, P30‐15), NKp46 (Biolegend, 29A1.4), NKG2D (Biolegend, 1D11), NKG2A (Biolegend, S19004C), CD94 (BD Biosciences, HP‐3D9), CD69 (Biolegend, FN50), TRAIL (Biolegend, RIK‐2), FasL (Biolegend, NOK‐1), GzmB (Biolegend, QA18A28), Perforin (Biolegend, dG9), and PE‐Labelled Human Mesothelin /MSLN (296‐580) Protein (Acro Biosystems, MSN‐HP2H5). The cells were resuspended in the DAPI (Sigma‐Aldrich) solution and analysed with BD LSRFortessa X‐20 cytometer (BD Biosciences). Flow cytometry data were analysed by the FlowJo software (Three Star, Ashland OR).

### 2.6 Assessment of CD107a, TNF‐α, and IFN‐γ by flow cytometry

MSLN CAR‐iNK cells or iNK cells were incubated with or without PMA/ionomycin (PMA/Ion) (MULTISCIENCES) or A1847 tumour cells (E:T = 1:3) for 2 h, followed by adding BFA/Monensin (MULTISCIENCES) for additional 2‐h incubation. After incubation, cells were stained with CD107a (Biolegend, H4A3), CD45 (Biolegend, HI30), CD3 (Biolegend, HIT3a), and CD56 (Biolegend, HCD56). FIX & PERM Kit (MULTISCIENCES) was used for fixation and permeabilization, followed by intracellular staining for IFN‐γ (Biolegend, 4S.B3) or TNF‐α (Biolegend, MAb11). The cells were analysed with BD LSRFortessa X‐20 cytometer (BD Biosciences). Flow cytometry data were analysed by the FlowJo software (Three Star, Ashland OR).

### 2.7 Cytotoxicity assay

MSLN CAR‐iNK cells or iNK cells (Effector, E) were cocultured with primary human ovarian cancer cells, A1847 or AGS tumour cell lines (Target, T) labelled with carboxyfluorescein diacetate succinimidyl ester (CFSE; Beijing BioRab Technology Co. Ltd.) in 96‐well plates for 4 h at respective E:T ratios (E:T = 0.2:1, 0.5:1, 1:1, 2:1). In serial target killing assay, MSLN CAR‐iNK cells or iNK cells were cocultured with CFSE‐labelled A1847 or AGS tumour cells for 12 h (Round 1) at E:T = 1:1. Fresh tumour cells were added into all wells and cocultured with the remaining effector cells for another 12 h (Round 2) at the same E:T ratio. This process was repeated for a third round (Round 3). Target cell death was assessed with flow cytometer (BD LSRFortessa X‐20 cytometer, BD Biosciences) by the percentage of DAPI in CFSE‐positive population. Flow cytometry data were analysed by the FlowJo software (Three Star, Ashland OR).

### 2.8 Cryopreservation and thaw

MSLN CAR‐iNK aliquots (5 × 10^7^ cells/mL) were suspended in freezing medium (CryoStor® CS10, animal component‐free, defined cryopreservation medium with 10% DMSO) and were frozen using a controlled‐rate‐freezing device (Thermo Scientific™ CryoMed™, 7455). Then, the frozen cells were preserved for 6 months in liquid nitrogen. Cryopreserved MSLN CAR‐iNK cells were rapidly thawed in water (37°C) and were dropwise transferred into NK cell medium. Cells were centrifuged at 300*g* for 5 min and resuspended in NK cell medium. The viabilities and counts of cells after thawing were determined using the automatic cell counter (Countstar Rigel S5) by mixing them with AO/PI (Count Star, RE10212) staining solution at a ratio of 1:1.

### 2.9 Construction of the ovarian cancer xenograft models and treatment with MSLN CAR‐iNK cells

NCG mice (NOD/ShiLtJGpt‐*Prkdc* ^em26Cd52^ *Il2rg* ^em26Cd22^/Gpt, GemPharmatech Co., Ltd.) were intraperitoneally injected with the luciferase‐expressing A1847 (A1847‐luci^+^) cells (2 × 10^5^ cells/mouse) to construct the ovarian cancer xenograft models on day −1. Bioluminescence imaging (BLI) (IVIS Spectrum PerkinElmer) was performed on these models to determine the total flux (p/s), and the models with similar total flux (p/s) were randomly divided into three groups (Tumour only, Tumour + iNK, and Tumour + MSLN CAR‐iNK) on day 0. These models were irradiated (2.25 Gy, Rad Source RS2000) before iNK cells or MSLN CAR‐iNK cells administration. iNK cells or MSLN CAR‐iNK cells (1.0 × 10^7^–1.5 × 10^7^ cells/mouse) were intraperitoneally injected on days 0 and 7, respectively. BLI was performed every week to trace the tumour cells. Mice suffering from heavy tumour burden were euthanized for ethical considerations.

## 3 RESULTS

### 3.1 Generation of MSLN CAR‐expressing hESCs

**FIGURE 1:**
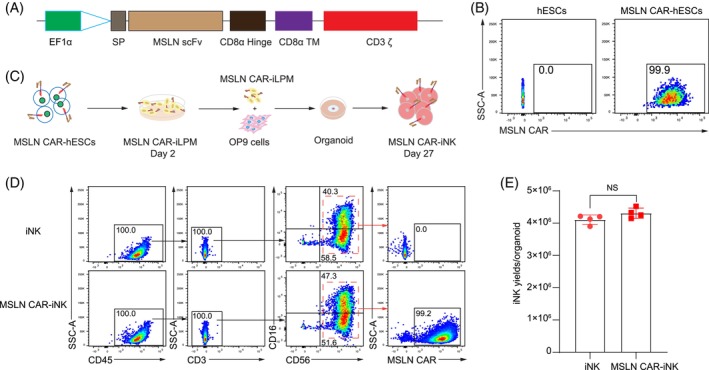
Generation of MSLN CAR‐expressing hESCs and induction of MSLN CAR‐iNK cells. (A) Schematic diagram of the transposon vector encoding the MSLN CAR. (B) Expression of MSLN CAR in hESCs. Representative flow cytometry plots showing the proportion of MSNL CAR‐expressing hESCs. (C) Schematic diagram of MSLN CAR‐iNK cell induction. (D) Immuno‐phenotypes of MSLN CAR‐iNK cells (CD45^+^CD3^−^CD56^+^CD16^+/–^MSLN CAR^+^) on day 27. (E) Statistics of yields of MSLN CAR‐iNK cells (day 27) induced from organoid aggregates. *n* = 4 organoids. NS, not significant (*p* > 0.05, two‐tailed independent *t*‐test, mean ± SD).

To achieve efficient and homogenous expression of MSLN CAR in iNK cells at a much lower cost, we engineered the MSLN CAR expression cassette into hESCs to construct the MSLN CAR‐expressing hESC (MSLN CAR‐hESC) line. MSLN CAR‐hESCs were further differentiated into MSLN CAR‐iNK cells. We designed the first‐generation of anti‐MSLN CAR comprising a scFv,^17^ a CD8α hinge, a CD8α transmembrane region (CD8 TM), and a CD3 ζ activation domain that is superior for NK cells in a comparative study^20^ (Figure 1A). The MSLN CAR cassette was integrated into the genomes of hESCs using the PiggyBac transposon system.^21^ The expression rates of MSLN CAR reached over 99% in hESCs after two rounds of sorting using a PE‐Labelled Human MSLN (296‐580) Protein that can be recognized by the MSLN CAR (Figure 1B). Thus, we successfully establish MSLN CAR‐hESCs.

### 3.2 Generation of MSLN CAR‐iNK cells from MSLN CAR‐hESCs

To induce MSLN CAR‐iNK cells, we adopted a reliable scale‐up method for generating human iNK cells from hPSCs.^19^ The unmodified hESCs were used as a control line to supervise any differentiation disruption caused by MSLN CAR engineering. The MSLN CAR‐expressing lateral plate mesoderm cells (MSLN CAR‐iLPM) were efficiently induced from MSLN CAR‐hESCs after 2‐day monolayer induction. On day 2, the MSLN CAR‐iLPM cells and OP9 cells were mixed to prepare organoid aggregates. The organoids were plated into the transwell for 25‐day NK cell induction (Figure 1C). Flow cytometry analysis revealed that over 99% of cells expressed MSLN CAR among the mature iNK (CD45^+^CD3^−^CD56^+^CD16^+/−^) cells (Figure 1D). On average, single organoids can output around 4.31 ± 0.16 million (mean ± SD) MSLN CAR‐iNK cells, which are comparable to iNK cells (4.11 ± 0.14 million, mean ± SD) induced from unmodified hESCs of the same genetic background (*p* > 0.05) (Figure 1E). Taken together, the MSLN CAR‐iNK cells are efficiently generated from MSLN CAR‐hESCs based on the organoid induction method we previously established.

### 3.3 Characterization of MSLN CAR‐iNK cells

**FIGURE 2:**
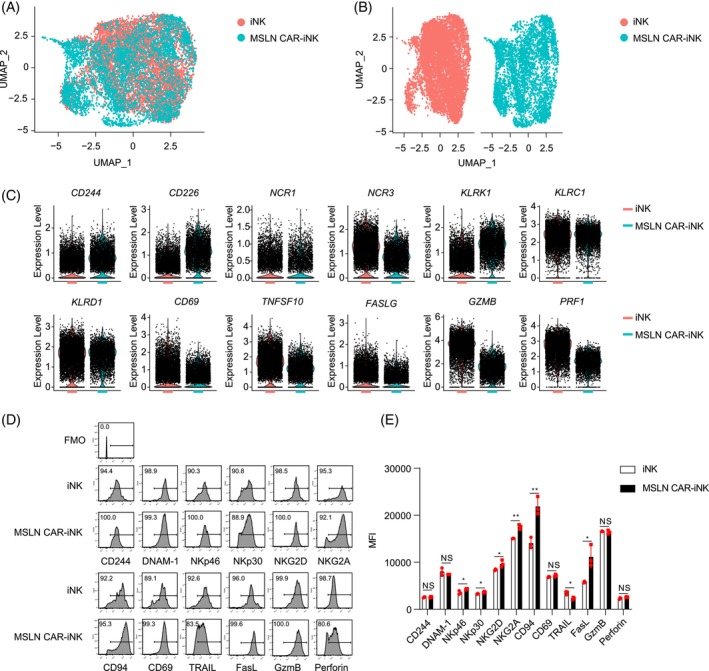
Molecular features of iNK cells and MSLN CAR‐iNK cells. (A) UMAP visualization of iNK cells and MSLN CAR‐iNK cells. (B) Projection of iNK cells and MSLN CAR‐iNK cells. (C) Violin plots showing the expression profiles of *CD244*, *CD226*, *NCR1*, *NCR3*, *KLRK1*, *KLRC1*, *KLRD1*, *CD69*, *TNFSF10*, *FASLG, GZMB*, and *PRF1* in iNK cells and MSLN CAR‐iNK cells. One point represents one cell. (D) Representative flow cytometry histograms showing the expression of CD244, DNAM‐1, NKp46, NKp30, NKG2D, NKG2A, CD94, CD69, TRAIL, FasL, GzmB, and Perforin. (E) Statistic analysis of the median fluorescence intensity (MFI) in (D). *n* = 3 repeats. NS, not significant (*p* > 0.05), *p < 0.05, **p < 0.01 (two‐tailed independent *t*‐test, mean ± SD).

To elucidate the molecular characteristics of MSLN CAR‐iNK cells generated by MSLN CAR hESCs, we performed 10× single‐cell RNA sequencing (scRNA‐seq) of MSLN CAR‐iNK cells and iNK cells. UMAP analysis showed similar patterns of transcriptomes between MSLN CAR‐iNK cells and iNK cells (Figure 2A, B). In detail, both iNK cells and MSLN CAR‐iNK cells expressed not only the classical activating receptor codings genes, such as *CD244*, *CD226*, *NCR1, NCR3*, and *KLRK1*, but also the inhibitory receptor coding genes, such as *KLRC1* and *KLRD1*.^22^ The MSLN CAR‐iNK cells also expressed the crucial effector molecules, including apoptosis‐related ligands (*TNFSF10* and *FASLG*),^23^ cytotoxic granules (*GZMB* and *PRF1*),^24^ and activating molecule (*CD69*) (Figure 2C).^25^ The expression patterns of these molecules at protein levels from flow cytometry analysis were consistent with mRNA levels from scRNA‐seq data (Figure 2D). We noticed that the expression levels of NK cell activation‐related proteins, including NKp30, NKp46, NKG2D, and FasL, were expressed higher in MSLN CAR‐iNK cells when compared to those in iNK cells (*p* < 0.05 or *p* < 0.01). In addition, the NKG2A/CD94 inhibitory receptor was also upregulated in MSLN CAR‐iNK cells (*p* < 0.05 or *p* < 0.01) (Figure 2E).

**FIGURE 3:**
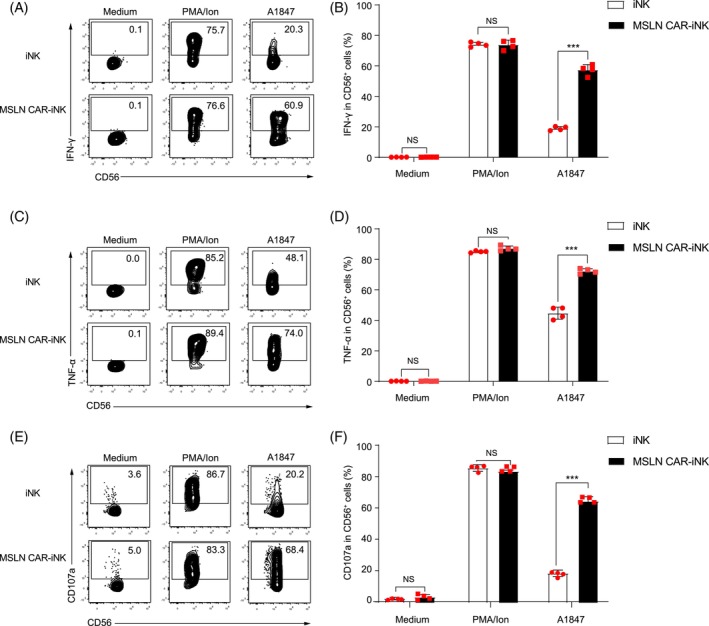
Evaluation of the secretion of IFN‐γ, TNF‐α, and expression of CD107a in MSLN CAR‐iNK cells. (A) Representative flow cytometry histograms showing the secretion of IFN‐γ by iNK cells or MSLN CAR‐iNK cells upon stimulation with PMA/ionomycin or A1847 tumour cells. (B) Statistic analysis of the secretion of IFN‐γ in (A). (C) Representative flow cytometry histograms showing the secretion of TNF‐α production by iNK cells or MSLN CAR‐iNK cells upon stimulation with PMA/ionomycin or A1847 tumour cells. (D) Statistic analysis of the secretion of TNF‐α in (C). (E) Representative flow cytometry histograms showing the expression of CD107a in iNK cells or MSLN CAR‐iNK cells following stimulation with PMA/ionomycin or A1847 tumour cells. (F) Statistic analysis of the expression of CD107a in (E). *n* = 4 repeats. NS, not significant (*p* > 0.05), ***p < 0.001 (two‐tailed independent *t*‐test, mean ± SD).

To elucidate the ability of secretion of IFN‐γ and TNF‐α, and the expression of CD107a in MSLN CAR‐iNK cells, we first stimulated MSLN CAR‐iNK cells and iNK cells with PMA/Ion. The results showed that MSLN CAR‐iNK cells have similar expression levels of IFN‐γ, TNF‐α, and CD107a compared with iNK cells (*p* > 0.05), which demonstrated that MSLN CAR‐iNK cells maintained the activation features (Figure 3A–F). As expected, MSLN CAR‐iNK cells exhibited elevated production of IFN‐γ and TNF‐α, and expression of CD107a compared to iNK cells when stimulated with A1847 (MSLN^+^) cells (*p* < 0.001), indicating MSLN CAR‐iNK cells can target the MSLN‐expressing A1847 cells (Figure 3A–F). In summary, these results demonstrate that MSLN CAR‐iNK cells express typical NK cell molecules akin to iNK cells. The MSLN CAR‐iNK cells exhibit a robust increase in the expression of IFN‐γ, TNF‐α, and CD107a upon stimulation by MSLN‐expressing tumour cells.

### 3.4 Revived MSLN CAR‐iNK cells exhibit ideal cytotoxic killing activity against A1847 tumour cells

**FIGURE 4:**
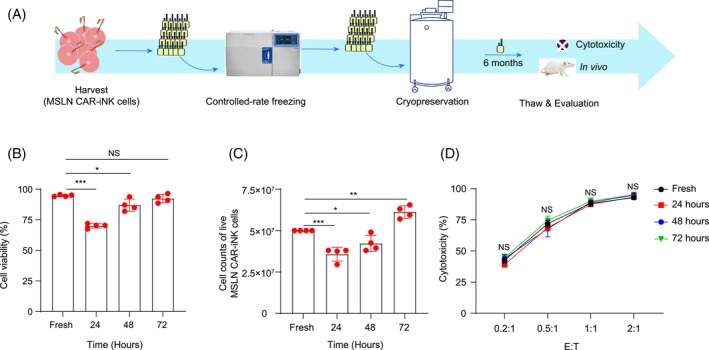
Evaluation of the viability and tumour‐killing ability of fresh and cryopreserved MSLN CAR‐iNK cells. (A) Schematics showing the design of evaluating the cytotoxicity of the cryopreserved MSLN CAR‐iNK cells. (B) Statistic analysis of the viability of MSLN CAR‐iNK cells at 24, 48, and 72 h post‐thaw. (C) Statistics analysis of the number of live MSLN CAR‐iNK cells at 24, 48, and 72 h post‐thaw. (D) Cytotoxicity analysis of the MSLN CAR‐iNK cells at 24 48, and 72 h post‐thaw against A1847 tumour cells at E:T = 0.2:1, 0.5:1, 1:1, 2:1. *n* = 4 repeats. NS, not significant (*p* > 0.05), *p < 0.05, **p < 0.01, ***p < 0.001 (one‐way ANOVA, mean ± SD).

To mimic off‐the‐shelf CAR‐iNK cell products for clinical applications, we used the clinical‐grade freezing medium (CryoStor® CS10, animal component‐free, defined cryopreservation medium with 10% DMSO) to cryopreserve the MSLN CAR‐iNK cells (5 × 10^7^ cells/mL). We kept the cells in liquid nitrogen over 6 months before the activity evaluation (Figure 4A). The viability and cytotoxic activity of the thawed MSLN CAR‐iNK cells were analysed after incubation for 24, 48, and 72 h. Despite the immediate viability of recovered cells after thawing is more than 90%, we still observed a 20%–30% loss of MSLN CAR‐iNK cells 24 h after thawing (*p* < 0.001). Nonetheless, an extended incubation of 24–48 h showed a significant recovery of total cell numbers (Fresh vs. 48 h group, *p* < 0.05; Fresh vs. 72 h group, *p* < 0.01) and viabilities (Fresh vs. 48 h group, *p* < 0.05; Fresh vs 72 h group, *p* > 0.05) (Figure 4B, C). Of note, the recovered cells and fresh cells exhibited no differences in cytotoxic killing activity against A1847 tumour cells (Figure 4D). Therefore, the cryopreserved MSLN CAR‐iNK cells show decent viability and cytotoxic activity after over 6 months of cryopreservation.

### 3.5 MSLN CAR‐iNK cells efficiently eliminate MSLN‐expressing tumour cells, and sustain cytotoxicity in consecutive tumour‐killing assays in vitro

**FIGURE 5:**
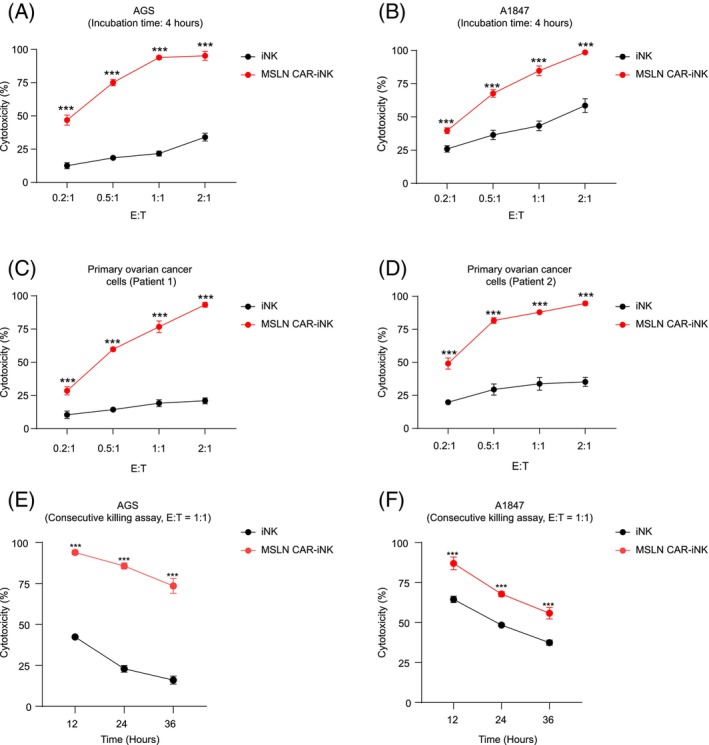
Evaluation of the tumour‐killing activities of MSLN CAR‐iNK cells in vitro. (A) Cytotoxicity analysis of iNK or MSLN CAR‐iNK cells against AGS tumour cells at the indicated effector to target ratios after 4 h incubation. (B) Cytotoxicity analysis of iNK cells or MSLN CAR‐iNK cells against A1847 tumour cells at the indicated effector to target ratios after 4 h incubation. (C), (D) Cytotoxicity analysis of iNK cells or MSLN CAR‐iNK cells against primary MLSN^+^ ovarian cancer cells isolated from ascites of two patients at the indicated effector to target ratios after 4 h incubation. (E), (F) Serial killing ability analysis of the iNK cells and MSLN CAR‐iNK cells. iNK cells and MSLN CAR‐iNK cells were co‐cultured with AGS (E) or A1847 (F) tumour cells for 12 hs per round (3 rounds) at the E:T ratio of 1:1. *n* = 4 wells. ***p < 0.001 (two‐tailed independent *t*‐test or Mann–Whitney U test, mean ± SD).

We subsequently performed tumour‐killing assays to validate the cytotoxicity of MSLN CAR‐iNK cells against tumour targets. Tumour cell lines with MSLN expression (human gastric cancer cell line, AGS; human ovarian cancer cell line, A1847) were targets for specific cytotoxicity assay. MSLN CAR‐iNK cells and iNK cells recovered 48 h after revival were used for tumour‐killing assay. Tumour cells (targets, T) were cocultured with either MSLN CAR‐iNK cells or iNK cells (effectors, E) at E:T = 0.2:1, 0.5:1, 1:1, 2:1 for 4 h. MSLN CAR‐iNK cells showed higher cytotoxic effects than unmodified iNK cells (Figure 5A, B). Additionally, primary tumour cells from two ovarian cancer patients were incubated with MSLN CAR‐iNK cells or iNK cells for 4 h. As expected, MSLN CAR‐iNK cells effectively killed primary ovarian tumour cells (Figure 5C, D). To further evaluate the sustained cytotoxicity of MSLN CAR‐iNK cells, we performed consecutive cell‐killing assays using AGS and A1847 tumour cells. MSLN CAR‐iNK cells or iNK cells were co‐cultured with AGS or A1847 cells at E:T = 1:1 for 12 h (Round 1). Fresh AGS or A1847 were added to the co‐culture wells containing residual MSLN CAR‐iNK cells or iNK cells for another 12 h (Round 2). The final round (Round 3) killing assay was performed as Round 2. The results demonstrated that MSLN CAR‐iNK cells exhibited robust serial tumour‐killing activity superior to iNK cells (Figure 5E, F). Collectively, MSLN CAR‐iNK cells show significantly enhanced cytotoxic activity against MSLN‐positive tumour cells.

### 3.6 MSLN CAR‐iNK cells suppress tumour growth in xenograft animals

**FIGURE 6:**
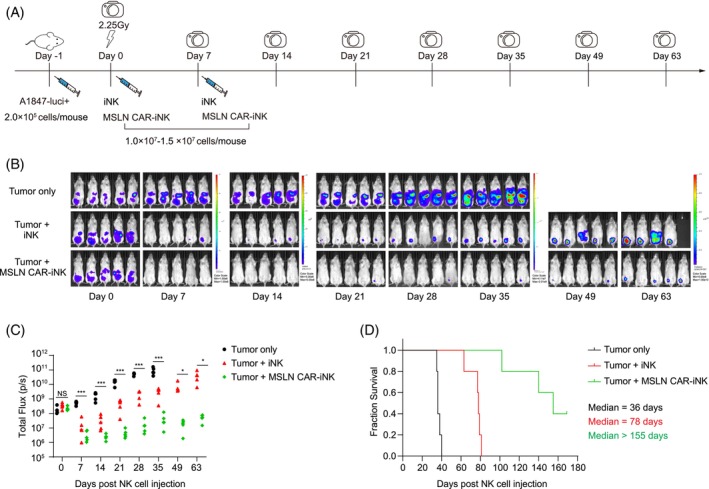
Evaluation of the tumour‐killing activity of MSLN CAR‐iNK cells in xenograft animals. (A) Diagram of A1847 cell xenogeneic NCG mice models for iNK cells and MSLN CAR‐iNK cells against tumour cells in vivo. Mice were treated intraperitoneally with iNK cells or MSLN CAR‐iNK cells on days 0 and 7. BLI was performed on days 0, 7, 14, 21, 28, 35, 49, and 63. (B) Bioluminescence images of the xenograft models (tumour only, tumour + iNK, and tumour + MSLN CAR‐iNK, *n* = 5 mice in each group). The radiance indicates tumour burden. (C) Statistics of the total flux (p/s) of the xenograft models *p < 0.05, ***p < 0.001 (*n* = 5 mice in each group, one‐way ANOVA and Two‐tailed independent *t*‐test). (D) Kaplan–Meier survival curves of the xenograft models. Median survival: Tumour only, 36 days; Tumour + iNK, 78 days; Tumour + MSLN CAR‐iNK, >155 days. *p* < 0.001 (Log‐rank test).

To further evaluate the therapeutic effects of the cryopreserved MSLN CAR‐iNK cells on tumour cells in vivo, we established a human ovarian cancer xenograft animal model by transplanting the luciferase‐expressing A1847 (A1847‐luc^+^) cells (2 × 10^5^ cells/mouse) into NCG (NOD/ShiLtJGpt‐*Prkdc* ^em26Cd52^ *Il2rg* ^em26Cd22^/Gpt) mice on day −1. Then, the cryopreserved iNK cells or MSLN CAR‐iNK cells were thawed and intraperitoneally injected into the tumour‐bearing animals (1.0 × 10^7^–1.5 × 10^7^ iNK cells/mouse) on days 0 and 7. BLI was performed weekly to capture the kinetics of tumour growth (Figure 6A). Indeed, the cryopreserved MSLN CAR‐iNK cells showed stronger tumour‐killing ability than iNK cells in xenograft animals. Meanwhile, the tumour burden of the tumour‐only group became increasingly severe, as indicated by the radiance and total flux (Figure 6B, C), and eventually, this group of mice needed ethical euthanasia due to the heavy tumour burden between days 35 and 40 post A1847‐luc^+^ cell injection. The cryopreserved MSLN CAR‐iNK cell‐treated mice survived significantly longer than the iNK cell‐treated mice and the tumour‐only mice (Tumour only: 36 days; Tumour + iNK: 78 days; Tumour + MSLN CAR‐iNK: >155 days; *p* < 0.001) (Figure 6D). In conclusion, these results show that the MSLN CAR‐iNK cells can efficiently suppress tumour development and prolong the survival of tumour‐bearing mice.

## 4 DISCUSSION

In this study, we provide a reliable approach for generating large‐scale and cryopreserved MSLN CAR‐iNK cells from hESCs. First, the hESCs were engineered to express MSLN CAR cassette. Following the hESC‐derived NK induction method we have previously reported,^19^ MSLN CAR‐iNK cells were efficiently produced in a short time window of 27‐day induction. The MSLN CAR‐iNK cells exhibit the typical expression patterns of activating receptors, inhibitory receptors, and effector molecules of NK cells. In the presence of tumour cells, the MSLN CAR‐iNK cells show increased secretion of IFN‐γ and TNF‐α, as well as elevated cytotoxic degranulation level (CD107a expression) compared with iNK cells. After 6‐month cryopreservation, we observed around 20%–30% loss of MSLN CAR‐iNK cells 24 h after revival. However, the total cell number and viability recover to over 90% with an extended 24–48 h of incubation. The MSLN CAR‐iNK cells effectively eliminate MSLN^+^ tumour cells in vitro, including the primary tumour cells from ovarian cancer patients. In particular, the cryopreserved MSLN CAR‐iNK cells suppress tumour growth and prolong the survival of human ovarian tumour animals.

The hESCs used in this study were established through a complete process in vitro and provided by the National Stem Cell Resource Center of China, in compliance with the related ethics and national legislation of China. In our study, single MSLN CAR‐hESC can produce 1078 ± 39 MSLN CAR‐iNK cells, which was comparable to the induction efficiency of unmodified hESCs.^19^ The MSLN CAR‐hESCs can output trillions of homogeneous MSLN CAR‐iNKs from one batch product with over 99% MSLN CAR expression in a GMP‐grade industrial facility. It reduces the variations in therapeutic effects caused by the heterogeneous NK cells derived from human primary tissues. Compared with the virus transduction method of delivering CAR elements into mature NK cells derived from cord blood or peripheral blood, this approach exhibited the advantages of low cost and homogeneity.

The MSLN CAR‐iNK cells also displayed higher expression levels of IFN‐γ, TNF‐α, and CD107a than iNK cells after stimulation with tumour cells, which is consistent with previous studies that CAR‐NK cells upregulate the expression levels of CD107a, IFN‐γ, and TNF‐α on conditions of stimulation by target cells.^8^, ^26^ Unlike traditional methods of expanding human tissue‐derived NK cells for CAR‐NK preparation, posing the risk of introducing potential severe side effects such as GvHD or cytokine release syndrome due to contaminating T cells,^27^, ^28^, ^29^ the hESC‐derived CAR‐iNK cells contain zero T cells.

We used a cryopreservation method tailored for CAR‐iNK cells to mimic real‐world clinical requirements. The cytotoxicity of the cryopreserved CAR‐iNK cells remains largely unaffected after 6‐month cryopreservation in clinical‐grade CS10 freezing medium. Additionally, the quantities and viabilities of the cryopreserved CAR‐iNK cells can be restored to pre‐cryopreservation levels post‐48‐h revival. This method has enabled us to overcome the challenges of NK cell cryopreservation, thereby facilitating an off‐the‐shelf product of CAR‐iNK cells. Moreover, cryopreserved NK cells provide sufficient time to allow evaluation of the potential risks of CAR‐iNK tumorigenesis, as CAR‐T cell‐caused secondary T‐cell tumours were reported after clinical administration.^30^ Recent research has reported that cryopreservation impairs the 3D migration and cytotoxicity of NK cells,^31^ which might compromise the therapeutic effects of cryopreserved CAR‐iNK cells and iNK cells in comparison to fresh cells.^19^ These findings are consistent with the dynamic changes observed in the quantity and viability of iNK cells post‐thawing. Therefore, in the context of cellular therapy applications, considering the characteristics of cryopreserved iNK cells, it is necessary to increase the original infusion dosage by 20%–30% to achieve the desired effective infusion dosage. Alternatively, cryopreserved iNK cells can be thawed and cultured for 48–72 h to offset the impacts.

In conclusion, our research provides an efficient, reliable, and low‐cost method for generating MSLN CAR‐iNK cells from hESCs. We provide evidence for the clinical translation of cryopreserved MSLN CAR‐iNK cells as a promising off‐the‐shelf cell product. This study offers insights into the clinical translation of hESC‐derived MSLN CAR‐iNK cells for ovarian cancer patients.

## AUTHOR CONTRIBUTIONS

Yanhong Liu, Min Zhang, and Xiaoyan Shen performed the core experiments and contributed equally to this work. Chengxiang Xia, Dehao Huang, Fangxiao Hu, Qi Zhang, Qitong Weng, Lijuan Liu, Yanping Zhu, Lei Wang, Jie Hao and Mengyun Zhang participated in multiple experiments. Chengxiang Xia, Dehao Huang, and Fangxiao Hu discussed the data and manuscript. Tongjie Wang and Jinyong Wang designed the project, discussed the data, and wrote the manuscript. Jinyong Wang provided the final approval of the manuscript.

## CONFLICT OF INTEREST STATEMENT

The authors declare no conflicts of interest.

## Data Availability

The data that support the findings of this study are available from the corresponding author upon reasonable request.
